# Encapsulation of grape seed oil in oil-in-water emulsion using multilayer technology: Investigation of physical stability, physicochemical and oxidative properties of emulsions under the influence of the number of layers

**DOI:** 10.1016/j.crfs.2024.100771

**Published:** 2024-05-16

**Authors:** Marziyeh Sepeidnameh, Ali Fazlara, Seyed Mohammad Hashem Hosseini, Mahdi Pourmahdi Borujeni

**Affiliations:** a Department of Food Hygiene, Faculty of Veterinary Medicine, Shahid Chamran University of Ahvaz, Ahvaz, Iran; b Department of Food Science and Technology, School of Agriculture, Shiraz University, Shiraz, Iran

**Keywords:** Grape seed oil, LBL emulsion, Stability, Oxidation

## Abstract

Many studies have shown that grape seed oil (GSO) is one of the vegetable fats that are plentiful in essential fatty acids and can be used as a fat substitute or to modify fat in food products to reduce saturated fatty acids. However, due to its low solubility and high sensitivity to oxidation, it is necessary to develop delivery systems that can distribute GSO in food more effectively. Recently, the preparation of emulsions using the layer-by-layer (LBL) method has many advantages in delivering lipid-soluble functional compounds. This research was used to check the formation of GSO oil-loaded primary, secondary and tertiary multilayer emulsions stabilized by mixture of anionic gelatin, cationic chitosan, and anionic basil seed gum (BSG) as the aqueous phase at pH 5, prepared using a layer-by-layer electrostatic deposition technique. Multilayer emulsions prepared by GSO and a mixture of gelatin, chitosan, and BSG as the aqueous phase at pH 5. Finally, the effect of the number of layers on the physicochemical properties (particle size, viscosity, turbidity, refractive index, and physical stability) and oxidative stability (peroxide value, thiobarbituric acid value, and fatty acid profile) during the storage time (30 days) at two temperatures 25 °C & 4 °C was investigated. Also, the zeta potential and Fourier transform infrared spectroscopy (FTIR) of mono-layer and multi-layer emulsions were investigated. The results revealed that by increasing the number of layers of multi-layer emulsion of GSO, the stability has improved. Thus, the tertiary emulsion has been more effective than the other two emulsions in maintaining the physicochemical characteristics and stability over time (P < 0.001). Morphological characterization and FTIR spectroscopy results confirmed that gelatin, chitosan, and BSG were successfully loaded into the LBL emulsions. This study can improve the original percept of multilayer emulsions and promulgate their potential applications for the entire encapsulation of essential fatty acids to enrich and prevent peroxide attack.

## Highlights

• Multilayer emulsions stabilized by gelatin and polysaccharides (chitosan and basil seed gum) were prepared at pH 5.
• Particle size, viscosity, turbidity, refractive index, peroxide and TBA index of emulsions increased during storage time.
• Increasing number of layers, increases the stability of emulsions and unsaturated fatty acids.
• Secondary layer formation in emulsion, reduced the oxidants diffusion between oil droplets and improved oxidative stability.

## Graphical abstract

**Figure:**
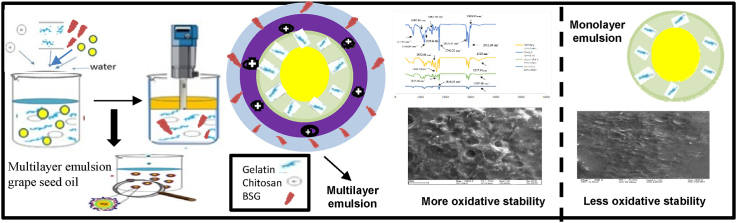

## 1 Introduction

The attention to GSO as a functional food product has increased, mainly due to its high levels of hydrophilic components, such as phenolic compounds, and lipophilic components, such as vitamin E, unsaturated fatty acids (UFAs), and phytosterols (Zhao et al., 2017). GSO contains 14–15% oleic acid (18:1), 68–76% linoleic acid (18:2), 0.6% alpha-linoleic acid (18:3), and about 1–18% saturated fatty acids (SFAs), palmitic acid (16:0) and stearic acid (18:0) (Diabetic et al., 2020). One of the constraints of the using GSO for human perception and in the food industry is the high sensitivity of n-6 fatty acids to oxidation (Boger et al., 2018). It is also necessary to expand beneficial delivery systems that can more effectively distribute GSO in liquid foods. Recently, the preparation of emulsions by using the layer-by-layer deposition method has many potential benefits for food applications, including better encapsulation of lipids, preventing the action of peroxides, controlling the release of bioactive compounds and the susceptibility to better the resistance of emulsions to processing and storage conditions (such as pH changes, salt addition, heating, freezing, and dehydration (Lesmes et al., 2010; Mundo et al., 2021; Acevedo-Fani et al., 2017). In this method, an oil-in-water emulsion is created by homogenizing oil, water, and a charged emulsifier. This emulsion is then mixed with a solution containing oppositely charged biopolymers, which are adsorbed to the surface of the oil droplets and form an additional coating (Tan et al., 2021). Non-covalent interactions (predominantly electrostatic) between oppositely charged biopolymers cause electrostatic deposition of one biopolymer on another biopolymer (emulsifier) and thus cause the formation of multilayer emulsions)Dickinson, 2019(. Several studies reported that multilayer emulsions provide better stability than conventional emulsions and give good encapsulation efficiency (Velez-Erazo et al., 2018; Li et al., 2021; Teo et al., 2017 & Kartal et al., 2017). Many types of proteins from vegetables and animals can be used as emulsifiers due to their amphiphilic character, polymeric structure, and electrical charge characteristics. Polysaccharides with strong electrostatic repulsion ability are used to create the subsequent layers) Burgos Diaz et al. (2016). In addition, polysaccharides are known as water retainers and surface membrane thickeners because of their hydrophilic power and high molecular weight (Damodaran, 2005). several charged natural biopolymers, such as gelatin/gum arabic have been used to form interfacial multi-layered delivery systems (Zhang et al., 2019), gelatin/gum arabic/tannic acid (Leiva-Vega et al., 2020), milk protein/alginate (Fioramonti et al., 2019), lactoglobulin/Arabic gum (Su et al., 2018), lactoferrin/alginate/ε-poly-L-lysine (Acevedo-Fani et al., 2017), whey protein hydrolysate/pectin (Tamm et al., 2016), whey protein Isolate/flaxseed gum/chitosan (Xu et al., 2016), whey protein/xanthan/locust bean (Griffin and Khouryieh, 2020).

Gelatins have been widely used as emulsifying agents in the food industry (Zhang et al., 2020). The properties of gelatins were dependent on the amino acid composition of gelatins, the hydrophobicity of amino acids in the gelatin polypeptide chains, and amino acid distribution on the gelatin surfaces, so emulsifying properties of gelatins were mainly dependent on surface hydrophobicity, and during the formation of emulsion, the hydrophobic regions of the protein can rapidly absorb to the surface of oil droplet (Xu et al., 2022). Chitosan is a very popular natural positively charged polysaccharide that is a linear copolymer of glucosamine and N-acetyl glucosamine connected through β-(1–4) glucosidic linkage) Evdokimov et al. (2015).(Furthermore, it has good functional properties and nutritional and physiological activities) Atarian et al. (2019). BSG by high viscous solution and shear thinning behavior is known to be a stabilizer, emulsifier, fat replacer, edible film, and controlling ice crystal growth (Hosseini-Parvar et al., 2015). BSG is an anionic heteropolysaccharide containing glucomannan, which has random coil conformation and is stable during heat and freeze/thaw treatments (Rafe and Razavi, 2013).

This study aimed to formulate single-layer (gelatin) and multilayer (gelatin/chitosan and gelatin/chitosan/BSG) emulsions containing GSO and to study the emulsion's stability and antioxidant activity during storage. Finally, the effect of interfacial layer number on the stability of GSO-loaded multilayer emulsions was discussed and proposed. Therefore, a basic understanding of the surface behavior and emulsification properties of systems containing gelatin as a protein biopolymer, BSG, and chitosan as a polysaccharide biopolymer with opposite charge will be helpful for improving the physicochemical, functional, and storage properties of emulsions containing GSO oil. However, the effect of interfacial layer number on the stability of the GSO-loaded multilayer emulsions remains unclear. It would fortify the basic theory of emulsions and provide potential advice for GSO-loaded multilayer emulsion development.

## 2 Materials and methods

### 2.1 Materials

GSO was purchased from (Pietro Coricelli CO) Italy, Edible Bovine Gelatine (Type B) (Bursa Jelatin Gida San. Ve Tic. A.S, Turkey), Chitosan (medium molecular weight) (SIGMA CHEMICAL CO., USA), Basil seed were obtained from a local market at Ahwaz city in Iran. The BSG was extracted according to Naji-Tabasi, et al. (2017). The chemical composition of BSG based on dry material (wt/wt) was: 5.80% ash, 1.35% protein, 5.7% moisture content, 81.60% total carbohydrate, 4.08% fat, 0.64% soluble sugars, and 0.83% starch. Deionized water was used for the preparation of all solutions.

### 2.2 Solution preparation

Gelatin solution was prepared by dispersing 1.0% (w/v) bovine bone gelatin granules into 100 mL deionized water, which was left for 30 min at room temperature to authorize the gelatin granules to swell. Then it was dissolved in a water temperature of 45 °C for 30 min on a magnetic stirrer, PIT300 (Pole Ideal pars Co, Iran) at 100 rpm. The aqueous solution of chitosan 2% (w/v) was prepared in 1% (v/v) of acetic acid and then stirred magnetically at 300 rpm for 1 h. The aqueous solution of BSG 0.6% (w/v) was dissolved in 100 ml of deionized water for 1 h with a magnetic stirrer, PIT300 (Pole ideal pars Co, Iran) at 100 rpm. The pH of the solutions solution was adjusted to 5–5.5 using 2.0 mol/L HCl and 2.0 mol/L NaOH.

### 2.3 Monolayer and multilayer emulsion preparation

#### 2.3.1 Monolayer emulsion preparation

Primary emulsion was obtained by blending 5 ml of GSO with 5 ml of prepared 1% gelatin solution at pH 5, using a homogenizer (SR30, top-Korea, Seoul, Korea) for 2 min at 12700 rpm.

#### 2.3.2 Multilayer emulsion preparation

To prepare secondary emulsions, 2 ml of chitosan solutions were added to 10 ml of primary emulsions under continuous stirring and followed by mechanical shearing with a homogenizer (SR30, top-Korea, Seoul, Korea) at a homogenization speed of 5800 rpm and a homogenization time of 60 s. All components were previously adjusted to pH 5, since the isoelectric points of type B bovine bone gelatin and chitosan were about 4.2, and 6.5, respectively at this pH biopolymers would be sufficiently charged to form electrostatic complexes. Tertiary emulsions were formed by diluting the secondary emulsions with 3 ml BSG 0.6% and followed by mechanical shearing with a homogenizer (SR30, top-Korea, Seoul, Korea) at a homogenization speed of 5800 rpm and a homogenization time of 60 s, then adjusted back to pH 5 using 1 M HCl. The zeta potential values of BSG-stabilized emulsions indicated that between pH 4 and 10, this gum is highly negatively charged, with values slightly decreased.

### 2.4 Evaluation stability monolayer and multilayer emulsions

To stabilize the multilayer emulsion, single-layer (gelatin), two-layer (gelatin-chitosan), and three-layer (gelatin-chitosan-BSG) emulsions were prepared at different pH (4–8), and according to the type of charge Emulsions at these investigated pHs and electrostatic interactions created between biopolymers in emulsions, their stability was checked after 48 h. Finally, the stability of all emulsions was studied after a month storage period.

### 2.5 Physico-chemical characteristics monolayer and multilayer emulsions

#### 2.5.1 Particle size and zeta potential

The droplet size and droplet size distribution (Span, equation 1) of the emulsions were measured by a particle size analyzer (PSA) (NanoTec 22, Germany). All emulsions were diluted before analysis with deionized water (pH 5) at 1:100 (v/v) to avoid multiple scattering effects. Span = (*D*v_0.9_ - *D*v_0.1_)/*D*v_0.5,_ where *D*_v0.1_, *D*_v0.5_, and *D*_v0.9_ indicate the droplet sizes, which 10%, 50%, and 90% of distribution lie below them, respectively.

The electrical charge of emulsions was examined using a Zetasizer Nano ZS device (Malvern Instruments, Worcestershire, U.K.). Emulsions were diluted 200-fold with 0.1 M acetate buffer before ζ-potential-measurements and analyzed in triplicate.

#### 2.5.2 Refractive index and turbidity

The turbidity of the samples was determined by using a UV–Vis spectrophotometer at 600 nm (SPECORD, Analytik Jena, Germany). It should be mentioned all emulsions produced were diluted 1:100 with deionized water at pH 5. The refractive index for prepared monolayer and multilayer emulsion were determined using Abbe’s type refractometer (OPTIKA Instruments, Italy) at 25 ± 1 °C. Samples were diluted in the ratio of 1:10 and all the measurements were taken in triplicates.

#### 2.5.3 Creaming index

Fresh monolayer and multilayer emulsions (10 mL) after preparation were poured into glass tubes tightly sealed with a plastic cap and then stored at room temperature. The height of the upper cream layer was measured after 7 days. The creaming index (%) is expressed as the ratio of the height of the upper cream layer (mm) to the height of the total emulsion (mm). CI (%) = Hs/H_T_ x 100%, where Hs represents the serum height and H_T_ represents the overall height of the emulsion. The creaming index (CI) determines the amount of serum and cream separated from the emulsion.

#### 2.5.4 Fourier transform infrared spectroscopy (FT-IR)

An FTIR spectrometer (Tensor II, Bruker, Germany) was applied to record the infrared spectra of the GSO, gelatin, chitosan, BSG, monolayer, and multilayer emulsions over the wavenumber range of 400–4000 cm^−1^ with a resolution of 4 cm^−1^ and 32 scans at 25 °C.

#### 2.5.5 Viscosity

The viscosity of the multilayer emulsions was measured at 12 RPM, 1 Spindle, and a temperature of 25 °C by Viscometer NDJ-8S (Shanghai Modern, China).

### 2.6 Scanning electron microscopy (SEM) of monolayer and multilayer emulsions

Scanning electron microscopy (SEM; LEO 1455VP, china) studied the surface morphology of the monolayer and multilayer emulsions of GSO. First, the emulsions were poured into a Petri dish and dried in a freeze-dryer. The sample was mounted on the specimen holder with aluminum tape and then coated with gold sputter coater.

### 2.7 Oxidative stability of multilayer emulsions

The oxidative stability of the multilayer emulsions was determined by measuring the primary (lipid hydroperoxides) and secondary (thiobarbituric acid reactive substances (TBARS)) lipid oxidation product concentrations. Changes in the lipid oxidation of multilayer emulsions were monitored during 28 day storage at 25 °C. Analyses were performed weekly.

#### 2.7.1 Measurements of peroxide value (PV)

According to the method of Shi et al. (2019), with some modification, Lipid hydroperoxides were measured by mixing 1 mL of multilayer emulsions with 5 mL of isooctane/2-propanol (3:1, v/v) The mixed solution was vortexed and centrifuged at 5000 rpm for 3 min 1 mL supernatant was mixed with 20 μL ammonium thiocyanate (3.94 M) and 20 μL ferrous solution (obtained from the supernatant of a mixture of 1 mL of 0.132 M BaCl2 and 1 mL of 0.144 M FeSO4). After that, 5 mL of methanol/1-butanol (2:1, v/v) was added to the organic solvent phase. The absorbance of the solution was measured at 510 nm for 30 min. Hydroperoxide concentrations were determined using a standard curve made from cumene hydroperoxide and measured as μM of CHP per gram of oil. All solutions were prepared freshly on a day-to-day basis for analysis.

#### 2.7.2 Measurements of thiobarbituric acid-reactive substances (TBARS)

TBARS test was used to determine the secondary lipid oxidation products according method of Sepeidnameh et al. (2018). 2 ml of emulsion samples were mixed with 2 mL of solution 2, which is comprised of 3 ml BHT alcoholic solutions (2 wt% in ethanol) and 100 ml solution 1. TCA (75 g), 12 M HCl (8.8 ml of), TBA (1.86 g), and double distilled water (414 g) were blended to prepare a mixed solution 1. The samples were placed in a boiling water bath for 15 min, cooled to room temperature, and then centrifuged for 10 min at 1000 g. then, the absorbance of the samples was measured at 532 nm with a UV–vis spectrophotometer. The TBARS concentrations were determined from a standard curve prepared using 1, 1, 3, 3-tetramethoxypropane.

### 2.8 Profile fatty acids of monolayer and multilayer emulsions

The analysis the changes in the profile of fatty acids over time were performed with a gas chromatograph (model GC-2550TG, Teif Gostar Fraz Co. Ltd, Iran) equipped with a flame-ionization detector (GC-FID) and a BP5 capillary column (25 m × 0.32 mm I. D. film thickness 0.5 μm). The column temperature was programmed as follows: initial oven temperature 40 C for 5 min, increasing to 100 C at 10 C min^−1^ and directly to 200 C at 20 C min^−1^, then holding for 1 min. The FID temperature was held at 260 C. High-purity hydrogen (99.999%) was used as the carrier gas with a flow rate of 1 mL min^−1^. Additional make-up gas was high-purity nitrogen with a flow rate of 60 mL min^−1^. The Oil extraction from the emulsion was determined using the method described by (Sepeidnameh et al., 2018).

### 2.9 Storage stability

The stability of all emulsions at two temperatures of 4 & 25 °C were compared in terms of appearance, turbidity, refractive index, viscosity, and particle size for 4 weeks, every 7 days. Also, the amount of creaming index was checked through centrifugation of the samples at two temperatures during 7 days of storage time.

### 2.10 Statistical analysis

All experiments were performed in 3 replicates, and the results were presented as mean ± standard deviation. Data were subjected to analysis of variance using LSD were used to analyze the data. α = 0.05 was considered as the basis of statistical judgment. Descriptive and analytical analysis of data were using SPSS 22 software and graphs were drawn using Excel 2013 software.

## 3 Results and discussion

### 3.1 Physicochemical properties of grape seed oil

The results of this study showed that the level of refract index for Pietro coricelli CO GSO is 1.472. The refract index of the sample was according to international standards. The refract index shows the oil quality and increases with the increase of compounds with higher molecular weight in oil and oxidation reaction. (Hussein and Abdrabba., 2015). The peroxide values and thiobarbituric acid of extracted oils from grape seeds were 1.7 meq/kgoil and 0.21 mg/kgoil respectively. Whereas peroxide is the first product from oil oxidation and TBA is a secondary product of oil oxidation. The characteristics of the fatty acids of oils are a measure of the amount of unique fatty acids in the oil and an essential factor in the quality of the oil. Fatty acid profile analyses of GSO are given in Table 1. Results showed, that the most fatty acids were related to linoleic acid (18:2) (46.63%) and oleic acid (18:1) (10.59%). Also, the percentage of unsaturated fatty acid (64.08%) was slightly higher than saturated fatty acid (35.92%). The composition of the fatty acids of the oil studied in the present study was similar to the composition of the fatty acids of the GSO studied in most research studies (Harbeoui et al., 2018; Wen et al., 2016; Lachman et al., 2015).

**Table 1:** Fatty acid composition of grape seed oil.

Fatty acid	%
**C**_**8:0**_	**1.40**
**C**_**10:0**_	**6.64**
**C**_**12:0**_	**3.69**
**C**_**14:0**_	**1.09**
**C**_**16:0**_	**1.20**
**C**_**17:0**_	**1.17**
**C**_**18:0**_	**20.23**
**C**_**18:1**_	**10.59**
**C**_**18:2**_	**46.63**
**C**_**18:3**_	**5.29**
**C**_**20:1**_	**1.57**
**C**_**22:0**_	**0.5**
**TSFA**	**35.92**
**TMUSFA**	**12.16**
**TPUSFA**	**51.92**

### 3.2 Stability and microstructure of multilayer emulsions

**Fig. 1:**
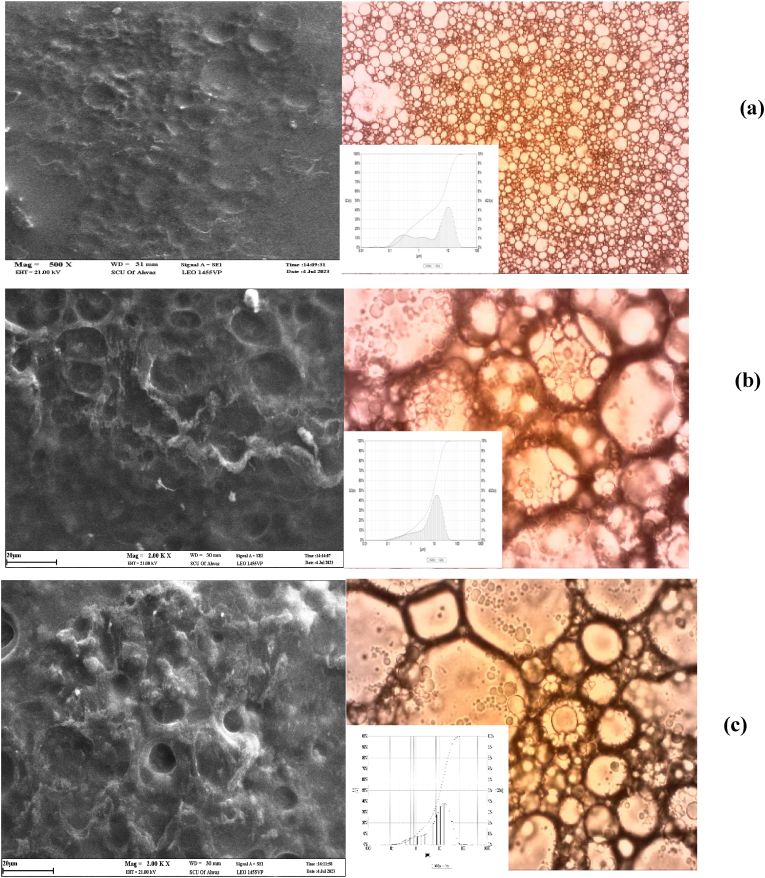
SEM and Optical Observation of primary (a), secondary (b) and tertiary (c) emulsions.

The effects of different pH (4, 5, 6, 7, or 8) on the stability of primary (gelatin), secondary (gelatin-chitosan), and tertiary (gelatin-chitosan-BSG) emulsions were investigated. The results showed that the highest stability for all emulsions was at pH 5, which may have been due to the type of charge of gelatin, chitosan, and BSG at this pH that created a good reduction in the electrostatic repulsion. It should be mentioned, that initially, the gelatin type B-coated lipid droplets in the primary emulsions were negatively charged, then interacted with positively charged chitosan and created the secondary emulsion stable. Tertiary emulsions stable were formed by added BSG, which shows that the anionic polysaccharide biopolymers (BSG) had adsorbed to the surfaces of the cationic lipid droplets and created electrostatic repulsion. In general, the stability of multilayer emulsion depends on various factors, including biopolymer type, droplet concentration, pH, and ionic strength (Tan2021). Morphological analysis (microstructure and layers of emulsions) of primary (gelatin), secondary (gelatin-chitosan), and tertiary (gelatin-chitosan-BSG) emulsions was performed using a scanning electron microscope. The external structure of the primary emulsion (Fig. 1, a) shows an irregular continuous surface with multiple elevations, which was probably related to the high amounts of oil covered inside and the leakage of GSO to the outside. The surface of the microstructures of the primary emulsion was smooth, while the surface of micro particles of secondary and tertiary emulsions was uneven; these prominent surfaces are related to the electrostatic interactions between protein and polysaccharide, which, with the increase in the number of multi-layer emulsion layers, these ridges were shown more (Fig. 1b and c). The holes or cavities created on the surface of all three emulsions were formed due to the sublimation of ice crystals, and a porous space around the oil droplets was created as a result of the evaporation of water during the freezing stage, in which ice is replaced by air. The ice crystals formed in the aqueous phase of emulsions at −18 °C may penetrate the oil droplets and disrupt their surface membrane, which increases the number of multilayer emulsion layers; these holes became more minor, which can indicate the more excellent stability of the multi-layer emulsion compared to the single-layer one, and therefore, with more GSO droplets coating, the microstructure remains intact. In general, it can be concluded that the formation of layers of biological biopolymers around oil droplets has reduced oil leakage from emulsion droplets and thus increases the encapsulation efficiency. Multilayer structures can help reduce the amount of free GSO content, because there will be less opportunity for the oil to come out from inside the seed to the surface of the particles. Similar results were reported in previous studies (Wang et al., 2022; Fang et al., 2019).

### 3.3 Physical properties of multilayer emulsions

**Fig. 2:**
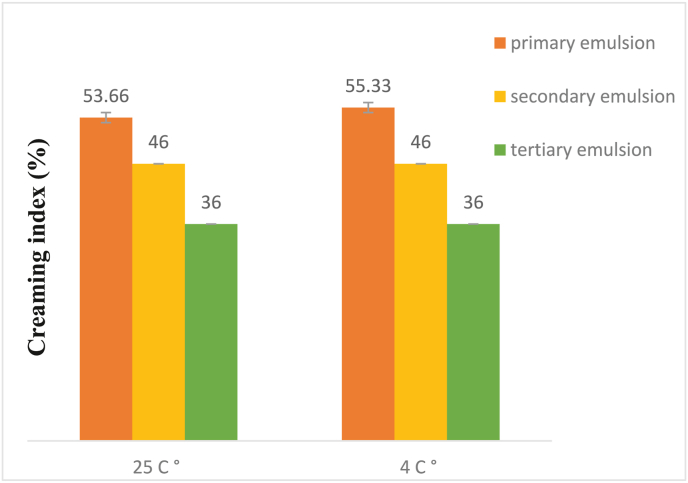
Changes in a creaming index (%) of multilayer emulsions after 7 days of storage at 4 °C and 25 °C.

The changes in the viscosity, Particle size, span, refractive index, and turbidity of emulsions at 4 °C & 25 °C during storage (28 days) are shown in Table 2. Concurrently, the creaming index after storage time (7 days) was also monitored in Fig. 2. Adsorption of a chitosan layer around oil droplets coated with gelatin increases the droplet size, and the electrostatic attraction between cationic gelatin and anionic chitosan is the driving force for absorption. The particle size of the droplets in primary, secondary, and tertiary emulsions at ambient temperature after the storage time increased from 2.04, 2.29, and 8.89 μm to 12.06, 13.17 and 20.09 μm, respectively, which indicates it increased with the increase in the number of layers. The change in mean droplet diameter by increasing the number of emulsion layers suggests that the thickness of the adsorbed biopolymers layer. The trend of particle size changes during the storage time in all emulsions had a significant difference (P < 0.001). The Average size of the droplets in emulsions increased at both temperatures (4 °C & 25 °C (during the storage time, and this increase was greater at ambient temperature (25 °C) than at refrigerator temperature (4 °C). This effect can mainly be attributed to the separation of polysaccharides from the continuous phase and being placed in the bulk phase, which has caused an increase in viscosity and then increased particle size. Results are in agreement with the previous reports that electrostatic reactions between biopolymers and the number of layers of multilayer emulsions are effective on the particle size of multilayer emulsions (Prichapan et al., 2021; Mundo et al., 2021; Yuliarti et al., 2019). Viscosity is a function of particle size and particle concentration, and the result of these two factors determines the decrease and increase of the final viscosity (Alexandre et al., 2016). Also, viscosity is a measurement of the resistance of a liquid to flow. In general, the viscosity on the first day of the storage time for the primary, secondary, and tertiary emulsions is 332.13, 525.63, and 426.46 (mPa·s), respectively. while at the end of the storage time at 4 °C temperature for the primary, secondary, and tertiary emulsions was 541.2, 533.66, and 530.1 (mPa·s), respectively, and at 25 °C temperature was 526.33, 542.3 and 511.26 (mPa·s), respectively. Viscosity increased during the storage period in all emulsions, increased was greater at 4 °C temperature than at 25 °C temperature. Moreover, the viscosity of the secondary emulsion was the highest compared to the other two emulsions, which is due to the high viscosity of chitosan compared to gelatin and BSG. A similar result regarding the high ability of chitosan to increase viscosity was reported by (Alexandre et al., 2016; Zhang et al., 2020). Changes in the absorbance of emulsions during storage at 25 °C and 4 °C were investigated and influenced by the number of layers. The turbidity after the storage time at 25 °C temperature for primary, secondary, and tertiary emulsions was 1.810, 1.830, and 1.304, respectively, and at 4 °C temperature was 1.590, 1.442, and 0.996 respectively, which has a significant difference (P < 0.001). On the first day of the storage period, the highest and lowest turbidity is related to the primary emulsion and the third emulsion, respectively. The reason for this trend is the increase in turbidity due to the increase in the number and size of particles) Shanmugam et al., 2012(. Other researchers have also reported that the turbidity is directly related to the amount of protein; in other words, the polysaccharide has interacted with the amount of protein present, and the additional polysaccharides through their free functional groups cause the complex to be charged, and by creating a strong electrostatic repulsion from its mass prevents and reduces the turbidity of emulsions (Yildiz et al., 2018; Hosseini et al., 2013). The results showed, during the storage time, the turbidity of the emulsions increased, which could be due to the increase in the size of the particles and approaching aggregation of droplets. Turbidity value of the multilayer emulsions at 25 °C higher than 4 °C temperature during storage time. This phenomenon is due to the increase of polysaccharide-protein hydrophobic interaction and the decrease of hydrogen bonding with increasing temperature (Ibrahim et al., 2022). Furthermore, the influence of temperature on the rate of turbidity of chitosan was rising at increasing temperatures (Marey, 2019). Similar trends have been reported by (Lin et al., 2021; Lee et al., 2022 & Niu et al., 2014). According to Table 2, the refractive index of the tertiary emulsion has a significant difference from the other two emulsions at both temperatures (4 °C & 25 °C) during the storage time (P < 0.001). While temperature has no significant effect on the refractive index of emulsions. The results showed that with the increase in the number of layers, the refractive index increased, which is since with the increase in the concentration of biopolymers, the number of particles in the solution increases, and when light enters the environment, it is refracted in contact with each particle, and as a result, the refractive index increases.

**Table 2:** Physicochemical properties of multilayer emulsion grape seed oil during storage at 25 °C and 4 °C.

Storage time(day)	emulsions	Particle size(μm)	Span(μm)	Viscosity (mPa·s)	turbidity	Refractive index
0	Primary emulsion(25 °C)	2.04 ± 0.011^Ec^	1.984 ± 0.9^Cc^	332.13 ± 0.581^Ec^	0.955 ± 0.001^Ea^	1.643 ± 0.000^Dc^
	Primary emulsion(4 °C)	2.04 ± 0.011^Ec^	1.984 ± 0.9^Ac^	332.13 ± 0.581^Dc^	0.955 ± 0.001^Ea^	1.643 ± 0.000^Cc^
	secondary emulsion(25 °C)	2.29 ± 0.011^Eb^	2.047 ± 1.2^Cb^	525.63 ± 0.218^Ca^	0.905 ± 0.001^Eb^	1.645 ± 0.001^Cb^
	secondary emulsion(4 °C)	2.29 ± 0.011^Eb^	2.047 ± 1.2^Ab^	525.63 ± 0.218^Ca^	0.905 ± 0.001^Eb^	1.645 ± 0.001^Bb^
	Tertiary Emulsion(25 °C)	8.89 ± 0.011^Ea^	2.615 ± 0.7^Aa^	426.46 ± 0.011^Db^	0.678 ± 0.001^Ec^	1.647 ± 0.000^Da^
	Tertiary Emulsion(4 °C)	8.89 ± 0.011^Ea^	2.615 ± 0.7^Aa^	426.46 ± 0.32^Db^	0.678 ± 0.001^Ec^	1.647 ± 0.000^Ca^
7	Primary emulsion(25 °C)	5.72 ± 0.011^Dc^	2.579 ± 0.13^Aa^	431.66 ± 0.88^Dc^	0.998 ± 0.000^Da^	1.648 ± 0.000^Cc^
	Primary emulsion(4 °C)	9.22 ± 0.011^Da^	1.742 ± 0.9^Bc^	523.4 ± 0.305^Cc^	0.993 ± 0.000^Db^	1.647 ± 0.000^Bc^
	secondary emulsion(25 °C)	9.52 ± 0.011^Db^	2.276 ± 1.2^Bc^	526.4 ± 0.240^Ca^	0.968 ± 0.000^Db^	1.650 ± 0.000^Bb^
	secondary emulsion(4 °C)	8.61 ± 0.011^Db^	1.984 ± 0.7^Bb^	526.06 ± 0.66^Ca^	0.982 ± 0.000^Db^	1.646 ± 0.000^Bb^
	Tertiary Emulsion(25 °C)	9.64 ± 0.011^Da^	2.506 ± 0.17^Bb^	472.66 ± 7.2^Cb^	0.869 ± 0.000^Dc^	1.652 ± 0.000^Ca^
	Tertiary Emulsion(4 °C)	8.58 ± 0.000^Db^	2.309 ± 1.1^Ba^	525.3 ± 0.15^Cb^	0.685 ± 0.001^Dc^	1.651 ± 0.000^Ba^
14	Primary emulsion(25 °C)	9.47 ± 0.011^Cc^	2.315 ± 0.19^Bb^	457.76 ± 0.392^Cc^	1.381 ± 0.001^Cb^	1.651 ± 0.001^Bb^
	Primary emulsion(4 °C)	11.56 ± 0.011^Ca^	0.906 ± 0.17^Ec^	524.6 ± 0.717^Cb^	1.21 ± 0.002^Ca^	1.653 ± 0.001^Ac^
	secondary emulsion(25 °C)	10.87 ± 0.011^Cb^	2.685 ± 1.1^Aa^	526.73 ± 0.26^Ca^	1.42 ± 0.000^Ca^	1.651 ± 0.000^Bb^
	secondary emulsion(4 °C)	10.70 ± 0.011^Cb^	1.675 ± 0.16^Ca^	528.53 ± 0.29^Ba^	0.987 ± 0.000A^Cb^	1.656 ± 0.001^Ab^
	Tertiary Emulsion(25 °C)	11.57 ± 0.011^Ca^	1.901 ± 1.8^Dc^	491.1 ± 0.49^Bb^	1.116 ± 0.001^Cc^	1.653 ± 0.000^Ca^
	Tertiary Emulsion(4 °C)	9.47 ± 0.013^Cc^	1.409 ± 0.4^Cb^	525.86 ± 0.08^Cb^	0.693 ± 0.003^Cc^	1.658 ± 0.000^Aa^
21	Primary emulsion(25 °C)	9.56 ± 0.000^Bc^	1.492 ± 0.21^Dc^	492.56 ± 1.27^Bc^	1.739 ± 0.001^Bb^	1.655 ± 0.001^Aa^
	Primary emulsion(4 °C)	12 ± 0.017^Ba^	1.570 ± 0.4^Ca^	527.9 ± 0.54^Bb^	1.51 ± 0.000^Ba^	1.657 ± 0.003^Aa^
	secondary emulsion(25 °C)	12.29 ± 0.006^Bb^	1.871 ± 0.2^Db^	534.96 ± 1.76^Ba^	1.763 ± 0.001^Ba^	1.656 ± 0.000^Aa^
	secondary emulsion(4 °C)	11.66 ± 0.011^Bb^	1.322 ± 0.21^Db^	530.2 ± 0.25^Ba^	0.992 ± 0.001^Bb^	1.657 ± 0.000^Aa^
	Tertiary Emulsion(25 °C)	15.98 ± 0. 11^Ba^	1.940 ± 0.9^Ca^	506.36 ± 1.41^Ab^	1.276 ± 0.001^Bc^	1.657 ± 0.000^Ba^
	Tertiary Emulsion(4 °C)	11.6 ± 0. 012^Bc^	1.187 ± 0.3^Dc^	527.13 ± 0.13^Bb^	0.817 ± 0.001^Bc^	1.658 ± 0.000^Aa^
28	Primary emulsion(25 °C)	12.06 ± 0.011^Ac^	1.123 ± 0.8^Ec^	526.33 ± 0.88^Ab^	1.81 ± 0.001^Ab^	1.658 ± 0.000^Aa^
	Primary emulsion(4 °C)	18.34 ± 0.011^Aa^	1.128 ±0 .07^Da^	541.2 ± 0.75^Aa^	1.59 ± 0.001^Aa^	1.657 ± 0.000^Ac^
	secondary emulsion(25 °C)	13.17 ± 0. 11^Ab^	1.496 ± 0.13^Ea^	542.3 ± 0.85^Aa^	1.83 ± 0.000^Aa^	1.658 ± 0.001^Aa^
	secondary emulsion(4 °C)	12.17 ± 0.011^Ab^	1.008 ± 1.2^Eb^	533.66 ± 0.88^Ab^	1.442 ± 0.001^Ab^	1.658 ± 0.000^Ab^
	Tertiary Emulsion(25 °C)	20.09 ± 0.103^Aa^	1.302 ± 1.1^Eb^	511.26 ± 0.89^Ac^	1.304 ± 0.001^Ac^	1.662 ± 0.001^Aa^
	Tertiary Emulsion(4 °C)	12.63 ± 0.012^Ac^	0.921 ± 1.2^Ec^	530.1 ± 0.44^Ac^	0.996 ± 0.000^Ac^	1.662 ± 0.001^Aa^

Different large letters indicate the difference in the storage time and Different small letters indicate the difference in the type emulsions at any temperature (P < 0.05).

Creaming is one of the gravitational separation forms which is an upward movement of droplets having lower density than the continuous phase (Kartal et al., 2017). Fig. 2, showed that the emulsion creaming stability was mainly dependent on the interfacial layer number, so it has decreased with the increase in the number of layers. Creaming index and emulsion stability are inversely related. In other words, the higher degree of creaminess of the primary emulsion compared to the other two emulsions can be attributed to the free movement of oil droplets in the system, so by increasing the number of layers due to the addition of polysaccharides (chitosan and BSG), the free movement of oil droplets in the system decreased and the viscosity created in the aqueous phase was sufficient to prevent oil droplets from collision and prevented the accumulation of droplets due to collision with each other. In addition, creaming of emulsions may have no significant effects after days of storage time. Type B gelatin used in this research has an isoelectric point of 4.2. At pH 5, which is above the isoelectric point of gelatin, the stable monolayer emulsion has a negative surface charge. The ζ-potential of the protein-coated oil droplets at pH 5 went from negative (−13.06 mV) to highly positive (+25.6 mV), when secondary emulsion is formed by adding chitosan, which can be attributed to the chitosan molecules were positive for all pH values studied, while the magnitude of the surface potential depending on the pH of the solution in which it is dissolved. The charge of chitosan remained relatively high by increasing until pH 5. If, the surface potential of chitosan decreased when the pH was raised. This event is due to the amino groups on the chitosan having pKa values around 6.5. These results indicated that the secondary emulsions tended to aggregate at higher pH values with little positive charge (Liu et al., 2019b). The optimal formulation of tertiary emulsions selected for the subsequent analyses contained 0.6% w/w BSG since these emulsions presented small droplet sizes, and strong positive ζ-potential (−30.8 mV).

### 3.4 FTIR analysis

**Fig. 3:**
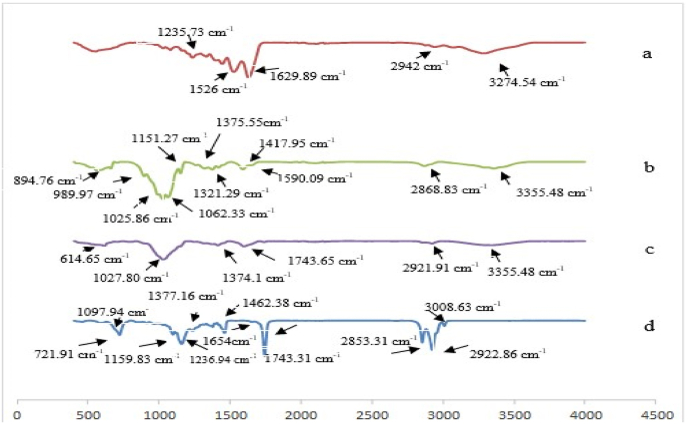
FTIR spectra of gelatin, chitosan, BSG, and GSO scanned at wavenumbers of 4000-400 cm^−1^.

**Fig. 4:**
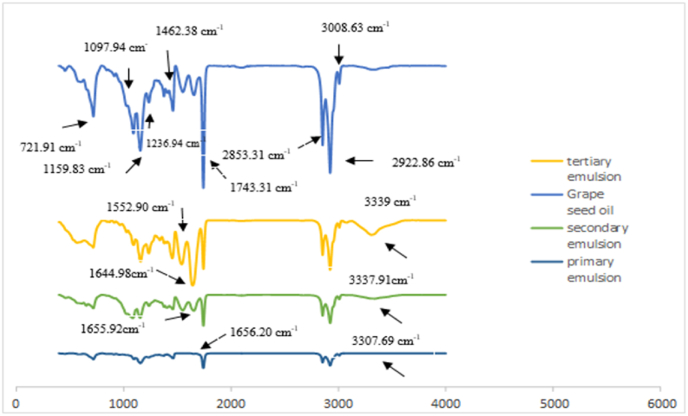
FTIR spectra of primary, secondary, and tertiary emulsions scanned at wavenumbers of 4000-400 cm^−1^.

FTIR spectra of gelatin, chitosan, BSG, GSO, and primary emulsion, secondary emulsion, and tertiary emulsion are shown in Fig. 3, Fig. 4. There are three different peaks with other edible oils in the FTIR spectrum of GSO, which are at 1147-1127 cm-^1^ (C–O stretching), 1106-1127 cm-^1^ (C–O stretching), and 650-802 cm-^1^, which is caused by C stretching. –C and O–H bending is observed (Akin et al., 2019). Functional peaks of GSO in the wave number of 1743.31 (stretching vibrations of carboxyl functional groups (bonded) C=O), 2853.31 cm-^1^ (methyl and methylene CH groups), 3008.63 cm-^1^ (C–H stretching vibration of cis double bond) (C=CH)), 2922.86 cm^−1^ (symmetric and asymmetric stretching vibration of methylene bond (CH2) and 1654 (cis C=C vibrations) were observed. This observation agreed well with the previous reports by (Berezin et al., 2018; Riyanta et al., 2020; Vladimir et al., 2021). The FTIR result of gelatin showed that the prominent peaks are located in the amide region. In the FTIR spectrum of gelatin used in this research, the amide A peak was observed at 3274. 54 cm^−1^, which indicates N–H stretching along with free O–H and hydrogen bonding. The peak of amide I at wave number 1629.89 cm^−1^ belongs to C=O stretching vibration with the help of C–N bond stretching vibration and N–H bond bending. This peak (between 1600 and 1700 cm^−1^) is the most essential and valuable for FTIR analysis of the second protein structure (Pradini et al., 2018). The peak of amide Π at 1526 cm^−1^ comes from NH bending vibration and C–N stretching vibration and indicates the vibration in the N–H and C–N planes of the amide bond or the vibration of the CH2 group (Hassan et al., 2021(, and the amide Ⅲ peak (1235.73 cm^−1^) indicates the combined peaks between C–N stretching vibrations and N–H deformation of bonds, as well as absorptions caused by mobile vibrations of CH2 groups (Hashim et al., 2010). Amide B was observed at 2942 cm^−1^, which is caused by the vibration of the C–H group of the protein, and this group is usually found in every organic compound (Almeida et al., 2012). In the FTIR spectrum of chitosan, the absorption peak at 3355.148 cm^−1^ is due to the O–H stretch, which overlaps with the NH stretch in the same region, and the absorption peak at 2868.83 cm^−1^ is the result of the asymmetric stretching vibrations of the C–H bond that occurs in this polysaccharide. Bending vibrations of amide Π at 1590.09 cm^−1^, C–N stretching of amide Ⅲ at 1321.129 cm^−1,^ and the bending vibration of CH2 was observed at 1417.95 cm^−1^ (Queiroz et al., 2014). The wave number peaks of 920–1190 cm^−1^ overlap C–N stretching vibrations and carbohydrate ring vibrations, which include peaks at 1151.27 cm^−1^ (absorption peak related to C–O–C asymmetric stretching), 1025. 86 cm^−1^ and 1062.33 cm^−1^ is related to (C–O stretching) and 989.97 cm^−1^ and 894.76 cm^−1^ (may be created from the amine group of chitosan). Also, the peak at 1375.155 cm^−1^ caused by the symmetric C–H bending vibrations of the CH2 group can be seen in chitosan spectroscopy (Cheng et al., 2020). The results of the FTIR spectrum of BSG confirm the presence of uronic acid in the wave number of 1400 and 1600 cm^−1^, and it is assigned to the symmetric and asymmetric stretching of C-OO, respectively (Naji-Tabasi et al., 2016). The peak appears at the wave number of 3343. 65 cm^−1^ is related to O–H stretching absorption due to intermolecular and intramolecular hydrogen bonds and shows the characteristic of stretching bonds of free hydroxyl groups (Tan et al., 2011). The peaks in the FTIR spectrum of BSG in the wavenumbers of 2921. 91, 1743.65, 1374.1, and 614.65 cm^−1^ are respectively related to C–H stretching vibration, CH2 bending vibration, stretching vibration of groups Carbonyl is CH3 bending vibration and N–O–O bending vibration (Naji-Tabasi et al., 2017; Ghumman et al., 2022). Also, the peak in the wave number of 1150–1990 cm^−1^, which occurred at 1027.80 cm^−1^, is attributed to C–O and C–O–C stretching bonds (Naji-Tabasi et al., 2016).

Based on these results of FTIR monolayer and multilayer emulsions, Characteristics of the peaks of GSO in the wavelengths of 721.91, 1097.94, 1159.83, 1236.94, 1377.16 and 1462.38, 1743.31, 2853.31, 3008.63 and 2922.86 cm^−1^, it appeared in the spectrum of three emulsions, which indicates the successful and complete encapsulation of GSO in the emulsions (Chen et al., 2018). In addition, no specific branching has been observed in the amide I peak, which indicates the uniform dispersion of biopolymers inside the emulsions; similar results have been reported by)Zhang et al., 2020). The carboxyl group attached to the double bond and the amide stretching vibration indicate the presence of peptide bonds in the surface layer (Li et al., 2020). Also, with the increase in the number of layers in multilayer emulsions, the intensity of peaks increased, which indicates the rise of hydrogen bonds formed between biopolymers. In other words, the intensity and power of peak amide I (1656.20 cm^−1^) and peak amide Π (1552.190 cm^−1^) were dependent on the interfacial layer number and the outer layer of emulsions: trilayer > bilayer > monolayer, This shows more stability of the tertiary emulsion. In addition, the peak of amide I in primary, secondary, and tertiary emulsions is 1656.20, 1655.92, and 1644.98 cm^−1^, respectively, and as reported by Liu et al., this decrease in wave number due to the increase in the number of layers indicates an increase in interaction electrostatic forces between biopolymers (Liu et al., 2021a). It should be noted that the hydroxyl peak in the primary emulsion has shifted from 3307.69 cm^−1^ to 3337.91 cm^−1^ and 3339 cm^−1^ with the increase in the number of layers, and these changes occur with the rise in hydrogen. This peak, which indicates the stretching absorption of OH due to intermolecular and intramolecular hydrogen bonds, shows the polymer stretching of OH and the stretching of N–H of amines and amides (Falsafi et al., 2022).

### 3.5 Oxidative stability analysis

**Fig. 5:**
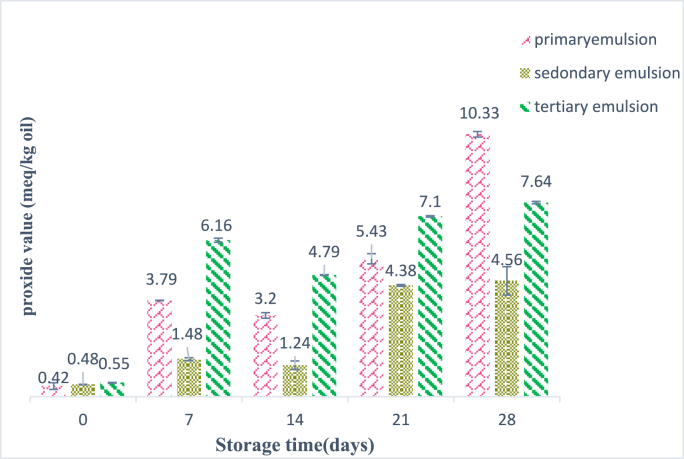
Changes in peroxide value (meq/kg oil) of multilayer emulsions during storage time and 25 °C.

**Fig. 6:**
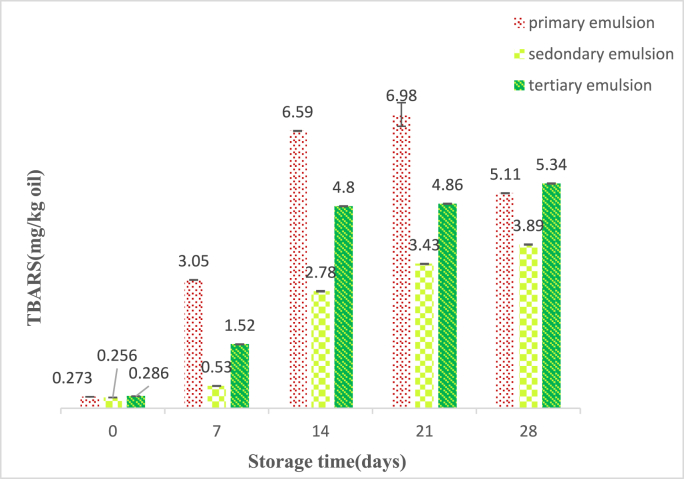
Changes in TBA (mg/kg oil) of multilayer emulsions during storage time and 25 °C.

Initially, unemulsification oil presented low values of PV (1.7meq/kg oil) and TBARS (0.21 mg/kg oil). The results showed that there was a significant difference in the changes of all three emulsions during the storage period (P < 0.001), and this trend was increasing (Fig. 5, Fig. 6). The peroxide value of the primary and the tertiary emulsions had significantly increased during the storage time, while this increase was slight in the secondary emulsion. The reason can be related to the positively charged chitosan that has covered the secondary emulsion and has created electrostatic repulsion with Fe^+^. In other words, by the increase in the number of emulsion layers, the oxidative stability increases, but because the tertiary emulsion is covered with BSG, which has a negative charge, it has absorbed more Fe^+^ than the other two emulsions (Ribeiro et al., 2023; Liu et al., 2021b). There was a significant difference (P < 0.05) between the TBARS value of multilayer and monolayer emulsions. Generally, the TBARS value in primary, secondary, and tertiary emulsions after the storage time was increased from 0.273, 0.256, and 0.286 mg/kg oil to 5.11, 3.89, and 5.34 mg/kg oil, respectively. As shown in Fig. 4, it was observed that the change trend of TBARS value in all emulsions during the storage time was increasing; the most increasing trend was related to the primary emulsion, and compared to the secondary emulsion and the tertiary emulsion, there was little change. It indicates that the chitosan-BSG coating is completely compact on the surfaces of the oil droplets. This layer of chitosan-BSG on the surfaces of the oil droplets effectively prevents the penetration of oxygen and other oxidation substances into the lipid cores and significantly reduces the initial oxidation of GSO in emulsions, which is agreement with the results obtained by (Sun et al., 2023), who showed that chitosan-pectin multilayer coatings effectively increase lipid stability against oxidative degradation. Also, in some days of storage time, the amount of peroxide and thiobarbituric acid value decreased, which can be caused by the decomposition of active hydroxides into other compounds. Eventually, the changing trend of TBARS value and peroxide value in all emulsions was consistent. As the authors reported, it has been shown that multilayer emulsions are less sensitive to oxidation than single-layer emulsions and are more stable (Wang et al., 2023; Mu et al., 2022; Espinosa-Andrews et al., 2023)

### 3.6 GC analysis

The fat content and the fatty acids profiles of multilayer emulsions during storage time and at 25 °C are summarized in Table 3. The PUFA/SFA ratio, known as the polyene index, is usually considered polyunsaturation and the oil's sensitivity to autoxidation. (Sepeidnameh et al., 2018). As shown in Table 3, with the increase in the number of layers, the amount of TUFA in emulsions increased and the amount of TSFA decreased. According to the results of (Chityala et al., 2016), it can be stated that the formation of a secondary layer can reduce the diffusion of peroxides between the oil droplets and hence improve oxidative stability. Polysaccharides generally show oxidative activity by donating H^+^ to free radicals and act as antioxidants that break the oxidant chain. Khouryieh also reported that the oxidative activity of an emulsion increases with the increase in the concentration of polysaccharides (Khouryieh et al., 2015). The ratio of PUFA/SFA in all emulsions decreased during the storage time and the highest level of PUFA/SFA was related to uncoated GSO (1.44%), and the lowest level was related to the primary emulsion of GSO (0.71%). Also, the results showed that with the increase in the number of layers, the ratio of PUFA/SFA increases and it can be estimated that multilayer emulsions are a more effective method than single-layer emulsions to preserve and stabilize essential unsaturated fatty acids and reduce the sensitivity to oxidation. Similar results have been reported by (Sanchez-osorno et al., 2022) about the inside coating of GSO. After 30 days of storage, the rate of omega-6 decreased, and the rate of omega-9 increased, which showed that the sensitivity of ω6 fatty acids to oxidation was higher than that of ω9 ones.

**Table 3:** Changes in the fatty acids profiles of monolayer and multilayer emulsions during storage time at 25 °C.

time(day)	emulsions	W_6_	W_9_	ƸMUFA	ƸPUFA	ƸSFA	ƸUFA	PUFA/SFA
**1**	Primary emulsion	37.13 ± 0.1^C^	6.08 ± 0.1^D^	7.31 ± 0.2^C^	38.59 ± 0.2^C^	54.1 ± 0.2^D^	45.90 ± 0.1^C^	0.71 ± 0.4^B^
**1**	Secondary emulsion	43.67 ± 0.8^A^	7.38 ± 0.3^B^	8.37 ± 0.4^B^	39.18 ± 0.2^B^	52.45 ± 0.1^E^	47.55 ± 0.4^B^	0.74 ± 0.6^C^
**1**	Tertiary Emulsion	40.96 ± 0.4^B^	5.43 ± 0.1^E^	6.28 ± 0.3^E^	42.22 ± 0.1^A^	51.50 ± 0.2^F^	48.50 ± 0.8^A^	0.81 ± 0.1^A^
**30**	Primary emulsion	31.31 ± 0.3^E^	9.14 ± 0.2^A^	9.94 ± 0.3^A^	32.71 ± 0.5^F^	57.35 ± 0.2^C^	42.65 ± 0.1^D^	0.57 ± 0.3^C^
**30**	Secondary emulsion	31.93 ± 0.2^E^	6.79 ± 0.1^C^	6.93 ± 0.2^D^	34.46 ± 0.1^E^	58.61 ± 0.2^B^	41.39 ± 0.2^E^	0.58 ± 0.1^C^
**30**	Tertiary Emulsion	32.49 ± 0.1^D^	6.91 ± 0.5^C^	6.91 ± 0.5^D^	35.06 ± 0.4^D^	59.03 ± 0.2^A^	41.97 ± 0.2^E^	0.59 ± 0.1^C^

• Different large letters indicate differences between emulsions during the storage period.

## 4 Conclusion

In summary, secondary and tertiary emulsions containing GSO could be successfully prepared by electrostatic deposition of cationic chitosan onto anionic gelatin-coated GSO droplets and anionic BSG onto cationic chitosan layers. The optimal conditions for emulsion preparation were 33.3% v/v GSO, 0.33 w t% Gelatin, 0.26 w t% CT and 0.12 w t% BSG. In general, the results showed that the most stability of the emulsions under the influence of different pH (4, 5, 6, 7, 8) was at pH 5, and with the increase in the number of layers of the multilayer emulsions of GSO-loaded, the stability increased. Also, the stability of emulsions at 4 °C temperature was higher than at 25 °C temperature. In fact, at pH 5, gelatin, chitosan, and BSG have negative, positive, and negative charges, respectively, and strong electrostatic interactions between biopolymers occurred, after which the LBL emulsions become stable. Therefore, the effects of the coating layers on lipid stability and profile fatty acid of emulsions were investigated. These results suggested that chitosan and BSG could form coating layers around lipid droplets, such as food-grade emulsions, which can inhibit lipid oxidation and increase the stability of essential unsaturated fatty acids. Eventually, this study can provide helpful information to understand the basic theory of multilayer emulsions. The preparation of multilayer emulsions of GSO can have potential applications in producing food products such as beverages and dairy products as a useful source of essential fatty acids, especially W6 and W9. Future research can focus on appling this emulsion system in foods as beneficial and the emulsion droplet stability during the gastric phase under the influence of interfacial layer number.

## CRediT authorship contribution statement

**Marziyeh Sepeidnameh:** Conceptualization, Visualization, Investigation, Writing – original draft, Preparation. **Ali Fazlara:** Supervision, Conceptualization, Methodology, Writing – review & editing. **Seyed Mohammad Hashem Hosseini:** Resources, Validation, Data curation. **Mahdi Pourmahdi Borujeni:** Software, Formal analysis.

## Declaration of competing interest

The authors declare that they have no known competing financial interests or personal relationships that could have appeared to influence the work reported in this paper.

## Data Availability

Data will be made available on request.
